# Strategies for repeat ablation for atrial fibrillation: A multicentre comparison of nonpulmonary vein versus pulmonary vein target ablation

**DOI:** 10.1111/jce.15441

**Published:** 2022-03-22

**Authors:** Daniel Mol, Mark J. Mulder, Rob Veenstra, Cornelis P. Allaart, Irene E. Hof, Michiel J. B. Kemme, Muchtiar Khan, Geert‐Jan P. Kimman, Gideon Mairuhu, Gijsbert S. de Ruiter, Giovanni J. M. Tahapary, Joris R. de Groot, Jonas S. S. G. de Jong

**Affiliations:** ^1^ Department of Cardiology OLVG Amsterdam The Netherlands; ^2^ Department of Cardiology Amsterdam University Medical Centres Amsterdam The Netherlands; ^3^ Noord‐West Hospital Group Department of Cardiology Alkmaar The Netherlands; ^4^ Department of Cardiology Flevohospital Almere The Netherlands

**Keywords:** atrial fibrillation, nonpulmonary vein target ablation, repeat ablation

## Abstract

**Introduction:**

Approximately 18% of patients with atrial fibrillation (AF) undergo a repeat ablation within 12 months after their index ablation. Despite the high prevalence, comparative studies on nonpulmonary vein (PV) target strategies in repeat AF ablation are scarce. Here, we describe 12 months efficacy of non‐PV and PV target ablations as a repeat ablation strategy.

**Methods:**

A multicentre retrospective, descriptive study was conducted with data of 280 patients who underwent repeat AF ablation. The ablation strategy for repeat ablation was at the operators' discretion. Non‐PV target ablation (*n* = 140) included PV reisolation, posterior wall isolation, mitral line, roofline, and/or complex fractionated atrial electrogram ablation. PV target ablation (*n* = 140), included reisolation and/or wide atrium circumferential ablation. Patients' demographics and rhythm outcomes during 12 months follow‐up were analyzed.

**Results:**

At 12 months, more atrial tachyarrhythmias were observed in the non‐PV target group (48.6%) compared to the PV target group (29.3%, *p* = .001). Similarly, a significantly higher AF and atrial tachycardia (AT) recurrence rate was observed after non‐PV target ablation compared to PV target ablation (36.4% vs. 22.1% and 22.9% vs. 10.7%). After adjustment, a significantly higher risk of AT recurrence remained in the non‐PV target group. Both groups significantly de‐escalated antiarrhythmic drug use; de‐escalation was more profound after PV target ablation. Patients with isolated PVs during non‐PV target ablation had a significantly higher risk for AF recurrence than those with reconnected PVs.

**Conclusion:**

Compared to PV target ablation, non‐PV target repeat ablation did not improve outcomes after 12 months and was independently associated with an increased risk for AT recurrences.

## 1 INTRODUCTION

Electrical isolation of the pulmonary veins (PVs) with ablation therapy results in a >99% reduction of atrial fibrillation (AF) burden in patients with paroxysmal AF.^1^, ^2^ PV isolation to maintain sinus rhythm is significantly more effective than antiarrhythmic drugs (AADs), but recurrence of AF is common, both after radiofrequency (RF) as after cryoablation.^3^, ^4^, ^5^

Despite improved durability of PV isolation with contemporary ablation techniques, 47% of patients have one or more reconnected PVs within 4–6 months after the index PV isolation procedure.^2^, ^6^ Approximately 18% of patients undergo repeat AF ablation within 1 year after the first AF ablation.^7^ In a recent multicentre randomized trial, electrophysiological mapping at the repeat AF ablation procedure revealed one or more reconnected PVs in up to 90% of the patients after contemporary AF ablation, likely suggesting the clinical need for reisolation.^8^

Besides PV triggers, non‐PV triggers have been advocated as an effective ablation target to improve rhythm outcomes.^9^ There is little data supporting non‐PV target ablation in addition to PV isolation during the initial procedure to improve AF‐free survival.^10^, ^11^, ^12^, ^13^ In fact, more extensive left atrial (LA) ablation has been associated with more atrial tachycardia (AT) recurrences.^13^

Reconduction after initial PV isolation has been associated with more atrial arrhythmia recurrences; it remains unclear whether non‐PV target ablation in addition to PV reisolation (or wider PV antrum isolation) should be preferred during a repeat AF ablation.^2^, ^14^, ^15^, ^16^, ^17^ This study compares non‐PV target with PV target ablation strategies during first repeat AF ablation in a multicentre retrospective cohort. We further sought to identify the prognostic implications of isolated PVs at repeat ablation in patients undergoing a non‐PV target ablation strategy.

## 2 METHODS

Patients undergoing their first repeat AF ablation between 2015 and 2019 were retrospectively included from OLVG Hospital Amsterdam and Amsterdam University Medical Centres location VUmc, Amsterdam, the Netherlands. Eligible patients had a previous AF (index) ablation and the primary indication for the repeat ablation was AF. Cases were performed with conventional RF or cryoballoon ablation for both the initial and repeat ablations. We excluded patients in whom AT was the primary indication for repeat ablation and patients who withdrew consent to use their data.

### 2.1 Ethical regulations

This study was conducted in full accordance with the Declaration of Helsinki (64th WMA General Assembly, Fortaleza, Brazil, October 2013) and the OLVG local ethics committee issued a waiver for an informed consent form. For study inclusion, a consent form to use clinical data was sent to all patients eligible for this study. Patients were asked to sign and return the consent form by mail (opt‐in). For patients who did not respond to our initial request, we tried to contact them by telephone. If all those attempts failed, data of the patients were included in the analyses following the Dutch Medical Treatment and Agreement Act.

### 2.2 Study objectives

We aimed to compare the clinical outcome of non‐PV target versus PV target ablation strategies during the first repeat AF ablation. Non‐PV target ablation was defined as PV reisolation in all patients with PV reconnection + additional LA ablation, including posterior wall isolation, mitral lines, roofline, and/or ablation of complex fractionated atrial electrograms (CFAEs). AF trigger ablation was not performed in this study. PV target ablation included reisolation of PV with or without wide antrum circumferential ablation or wide antrum circumferential ablation in case of already isolated PVs.

The primary outcome was the reported freedom of any atrial tachyarrhythmia at 12 months after the repeat AF ablation.^18^ Secondary outcomes included (i) freedom of AF and AT (including focal, micro, and macro re‐entrant ATs) at 12 months, (ii) the reported freedom of any atrial tachyarrhythmia, AF, and AT after repeat ablation in patients with reconnected PVs, (iii) the prognostic implications of isolated PVs in patients undergoing non‐PV target strategy at first repeat ablation on 12 months outcomes, (iv) AADs usage before repeat AF ablation and at 12 months follow‐up, (v) AF‐related symptoms before repeat AF ablation and at 12 months follow‐up, and (vi) procedural complications within 30 days.

### 2.3 Ablation procedure

The repeat AF ablation strategy and modality was at the operators' discretion and reflected the standard of care. Ablation modalities for repeat ablation included conventional RF (contact and noncontact force, multiple vendors) or the second‐generation cryoballoon (Medtronic). PV reisolation was defined as focal touch‐up ablation of reconnected PVs until entry and exit blocks were confirmed. Wide antrum circumferential ablation in patients with isolated or reconnected PVs increased the lesion size around the PV ostia and was considered a PV target strategy. Posterior wall isolation consisted of a roofline between the left and right superior PVs and a posterior line between inferior PVs. A mitral line was defined as any ablation line between the mitral valve annulus and a conduction barrier such as left inferior PV, right superior PV, or posterior ablation line. CFAE ablation was defined as ablation of fractionated electrograms with two or more deflections, continuous deflection of a prolonged electrogram, and/or electrograms with an average cycle length <120 ms over a 10‐s period.^19^

### 2.4 Follow‐up

Patients underwent routine clinical care follow‐up via referring hospitals. All available rhythm recordings during the 12 months follow‐up were retrieved from the medical records and analyzed for the current analysis. Recurrence of any atrial tachyarrhythmia was defined as an AF or AT episode lasting >30 s and documented on an electrocardiographic recording, including electrograms retrieved from cardiac implantable electronic devices.^18^ AF and AT recurrences were also assessed separately. A 90‐day blanking period was used for arrhythmia‐free survival. Atrial tachyarrhythmias that occur within this period were excluded for outcome analysis.^18^

### 2.5 AAD use

For a clarification purpose, we categorized the AAD use data as Grade 1—patients who used beta‐blockers or calcium antagonists (Vaughan Williams Class II or Class IV AAD) or no ADD, Grade 2—patients who used Class I AAD or sotalol, and Grade 3—patients who used amiodarone. De‐escalation of AAD use was defined as the discontinuation of AAD or the use of a lower grade AAD than before the procedure.

### 2.6 Echocardiographic definitions

LA dimensions and left ventricle (LV) function were interpreted as normal, mildly, moderately, or severely enlarged/impaired as per guideline recommendations.^20^ If discrete measures were not available, we used qualitative descriptions of LA dimensions and LV function.

### 2.7 Statistical analysis

Normally distributed variables are presented as mean ± standard deviation and non‐normal data as the median and interquartile range. Parametric *t* test, nonparametric test, and *χ* ^2^ test were performed for comparative analyses between groups. Multivariate logistic regression analysis was performed to adjust for confounding baseline characteristics. Covariates were included in the multivariate analysis if they changed the exposure coefficient by >10%.^21^ In the case of >1 covariates per 10 events, variables were excluded based on the lowest Akaike information criteria (AIC) to avoid overfitting. The excluded covariates and the change in AIC are reported. Only one patient had long‐standing persistent AF and was excluded from the regression model. Sensitivity analysis was performed with inverse propensity weighting to examine the robustness of the multivariate regression model on any atrial tachyarrhythmia, AT, and AF recurrence. The inverse propensity weights were calculated with the baseline characteristics and added to a logistic regression model. General estimation equations for continuous data and for correlated multinomial response were used to compare outcomes on AAD grade and AF‐related symptoms. All the statistical analyses were performed using R studio (version 1.4.1103).

## 3 RESULTS

### 3.1 Population

Two hundred and eighty‐six patients undergoing repeat AF ablation were eligible for this study, of whom 280 (98%) were included. Four patients were excluded because they refused consent, and two other patients died before the end of the 90‐day blanking period. The baseline clinical characteristics are summarized in Table 1.

**Table 1:** Baseline characteristics

	Overall (*n* = 280)	Non‐PV targets (*n* = 140)	PV targets (*n* = 140)	*p* Value
Age	62.8 ± 8.9	65.5 ± 8.1	60.0 ± 8.8	<0.001
Male	179 (63.9)	81 (57.9)	98 (70.0)	0.047
Body mass index	27.1 ± 4.2	27.0 ± 4.2	27.2 ± 4.1	0.774
CHA_2_DS_2_ VASc	1.6 ± 1.4	2.0 ± 1.3	1.2 ± 1.3	<0.001
Congestive heart failure	44 (15.7)	30 (21.4)	14 (10.0)	0.014
Hypertension	133 (47.5)	78 (55.7)	55 (39.3)	0.009
Diabetes	18 (6.4)	9 (6.4)	9 (6.4)	1.000
Stroke	30 (10.7)	18 (12.9)	12 (8.6)	0.334
Vascular disease	34 (12.1)	21 (15.0)	14 (10.0)	0.278
COPD	14 (5.0)	10 (7.1)	4 (2.9)	0.170
Atrial fibrillation type				<0.001
Paroxysmal atrial fibrillation	192 (68.6)	72 (51.4)	120 (85.7)	
Persistent atrial fibrillation	87 (31.1)	67 (47.9)	20 (14.3)	
LS persistent atrial fibrillation	1 (0.4)	1 (0.7)	0	
EHRA class	2.5 ± 0.6	2.6 ± 0.6	2.4 ± 0.6	0.003
Echocardiography				
Left atrium enlargement	(*n* = 276)	(*n* = 136)		0.040
Normal	98 (35.5)	39 (28.7)	59 (42.1)	
Mildly enlarged	61 (22.1)	31 (22.8)	30 (21.4)	
Moderately enlarged	64 (23.2)	32 (23.5)	32 (22.9)	
Severely enlarged	53 (19.2)	34 (25.0)	19 (13.6)	
Left ventricle function				0.184
Normal	235 (83.9)	111 (79.3)	124 (88.6)	
Mildly impaired	27 (9.6)	17 (12.1)	10 (7.1)	
Moderately impaired	13 (4.6)	8 (5.7)	5 (3.6)	
Severely impaired	5 (1.8)	4 (2.9)	1 (0.7)	
Medications				
Beta‐blocker	150 (53.6)	82 (58.6)	68 (48.6)	0.119
Class I antiarrhythmic drugs	93 (33.2)	43 (30.7)	50 (35.7)	0.447
Sotalol	69 (24.6)	40 (28.6)	29 (20.7)	0.166
Amiodarone	27 (9.6)	16 (11.4)	11 (7.9)	0.418
Calcium antagonist	65 (23.2)	27 (19.3)	38 (27.1)	0.157
Digoxin	4 (1.4)	2 (1.4)	2 (1.4)	1.000
Non‐vitamin K oral anticoagulation	187 (66.8)	90 (64.3)	97 (69.3)	0.447
Vitamin K oral anticoagulation	93 (33.2)	50 (35.7)	43 (30.7)	0.447
Previous ablation				
First ablation modality				0.011
Conventional RF	166 (59.3)	72 (51.4)	94 (67.1)	
Cryoballoon	114 (40.7)	68 (48.6)	46 (32.9)	
Posterior wall isolation	11 (3.9)	11 (7.9)	0	<0.001
Roofline	2 (0.7)	2 (1.4)	0	0.498
CFAE	4 (1.4)	3 (2.1)	1 (0.7)	0.622
Mitral line	2 (0.7)	2 (1.4)	0	0.498
Cavotricuspid isthmus ablation	54 (19.3)	23 (16.4)	31 (22.1)	0.289

*Note*: Non‐PV target ablation was defined as PV reisolation with additional LA ablation, and PV target ablation as PV reisolation with or without wide antrum circumferential ablation. Mean and standard deviation (±) and number (%).

Abbreviations: CFAE, complex fractionated atrial electrogram; CHA2DS2 VASc, congestive heart failure, hypertension, age (≥75, doubled), diabetes, stroke (doubled), vascular disease, age (≥65), sex); COPD, chronic obstructive pulmonary disease; EHRA, European Heart Rhythm Association; LA, left atrial; LS, long‐standing; PV, pulmonary vein; RF, radiofrequency.

Non‐PV target and PV target ablation were both performed in 140 (50%) patients. Patients undergoing non‐PV target ablation were older (65.5 ± 8.1 vs. 60.0 ± 8.8 years, *p* < .001), had a higher CHA_2_DS_2_VASc (congestive heart failure, hypertension, age (≥75, doubled), diabetes, stroke (doubled), vascular disease, age (≥ 65), sex) score (2.0 ± 1.3 vs. 1.2 ± 1.3, *p* < .001), and a higher incidence of severe LA enlargement (25.0% vs. 13.6%, *p* = .040) than those in the PV target group. Persistent AF was more frequent in the non‐PV target group compared to the PV target group (47.9% vs. 14.3%, *p* < .001) (Table 1). Conventional‐RF was used for the index ablation procedure in 166 (59.3%) patients (non‐PV target 51.4% vs. PV target 67.1%, *p* = .011) (Table 1).

### 3.2 Repeat AF ablation

Repeat ablation was predominantly performed with conventional RF (*n* = 275, 98.2%), whereas only five patients (1.8%) were treated with cryoballoon, all in the PV target group. Contact force‐sensing catheters were used in 112 (80.0%) patients and 108 patients (77.1%) in the non‐PV target and PV target group, respectively. Electrophysiological mapping before repeat ablation revealed that all PVs were isolated in 72 (25.7%) patients (non‐PV target 50.7% vs. PV target 0.7%, *p* < .001) (Table 2). Non‐PV target ablation strategy included PV re‐isolation in 71 (50.7%), posterior wall isolation (*n* = 90, 64.3%), mitral line (*n* = 46, 32.9%), roofline (*n* = 24, 17.1%), and CFAE ablation (*n* = 63, 45.0%) (Table S1). Complete conduction block across the linear lesion set was not obtained in 7/124 patients (5.7% of posterior wall isolation and 10% of mitral line). The PV target ablation strategy included focal reisolation (*n* = 115, 82.1%) and wide atrium circumferential ablation (*n* = 25, 17.9%) (Table S1). An AT was induced with pacing or started spontaneously during the repeat AF ablation in 15 patients (11 of non‐PV target and 4 of PV target group, *p* = .111). Cavotricuspid isthmus ablation was performed in 77 patients (27.5%), and accounted for 29.3% of non‐PV target and 25.7% of PV target group, *p* = .592.

**Table 2:** Procedural characteristics and follow‐up outcomes

	Overall (*n* = 280)	Non‐PV targets (*n* = 140)	PV targets (*n* = 140)	*p* Value
Procedural characteristics				
Modality				0.060
Conventional RF	275 (98.2)	140 (100.0)	135 (96.4)	
Cryoballoon	5 (1.8)	0	5 (3.6)	
Contact force	220 (78.6)	112 (80.0)	108 (77.1)	0.921
All PVs isolated	72 (25.7)	71 (50.7)	1 (0.7)	<0.001
Cavotricuspid isthmus ablation	77 (27.5)	41 (29.3)	36 (25.7)	0.592
Follow‐up				
Recurrence of ATa at 12 months	109 (38.9)	68 (48.6)	41 (29.3)	0.001
Recurrence of AF at 12 months	82 (29.3)	51 (36.4)	31 (22.1)	0.013
Recurrence of AT at 12 months	47 (16.8)	32 (22.9)	15 (10.7)	0.011
EHRA class	1.7 ± 0.8	1.9 ± 0.8	1.5 ± 0.6	<0.001

*Note*: Non‐PV target ablation was defined as PV reisolation with additional LA ablation, and PV target ablation as PV reisolation with or without wide antrum circumferential ablation. Mean and standard deviation (±) and number (%).

Abbreviations: AF, atrial fibrillation; AT, atrial tachycardia; ATa, atrial tachyarrhythmia; EHRA, European Heart Rhythm Association; PV, pulmonary vein; RF, radiofrequency.

### 3.3 Safety

Sixteen (5.7%) procedural complications occurred in 15 patients (nine in non‐PV target and six in PV target, *p* = .600), including vascular groin complication (*n* = 3, 1.1%), cardiac tamponade (*n* = 6, 2.1%), exacerbation of heart failure (*n* = 2, 0.7%), pericarditis (*n* = 2, 0.7%), hematuria after urinary catheter placement (*n* = 2, 0.7%), and complete heart block (*n* = 1, 0.4%).

### 3.4 Follow‐up

**Figure 1:**
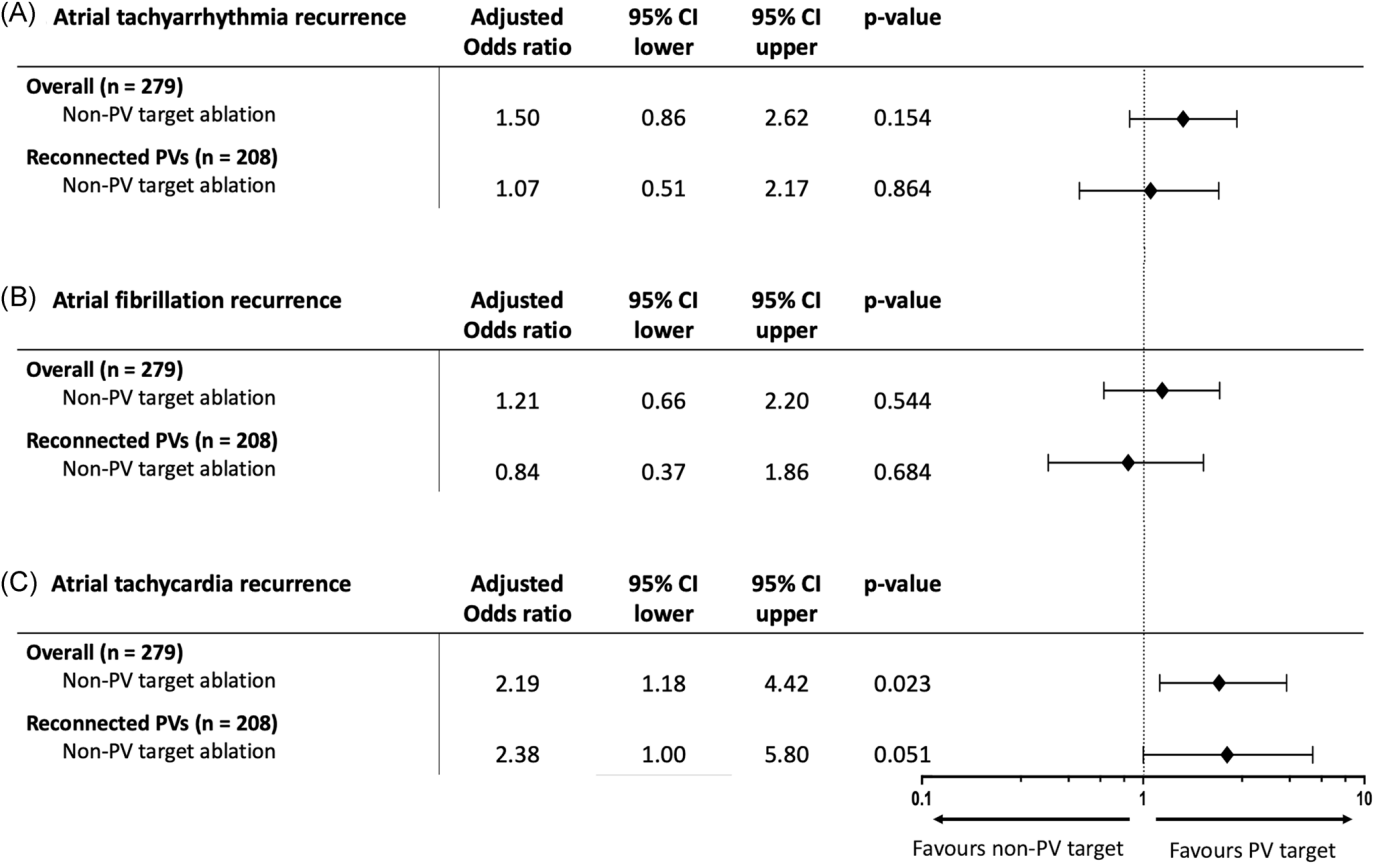
Arrhythmia recurrence risk of patients who underwent non‐pulmonary vein (PV) target ablation. This figure presents the atrial tachyarrhythmia (A), atrial fibrillation (B), and atrial tachycardia recurrence risk. Here, the odds ratio displays the recurrence risk of non‐PV target ablation versus PV target ablation strategy. Second, the odds ratio display the recurrence risk of non‐PV target versus PV target ablation strategy in patients with reconnected PVs before repeat AF ablation. Non‐PV target ablation was defined as PV reisolation with additional left atrial ablation, and PV target ablation as PV reisolation with or without wide antrum circumferential ablation. Of note, one patient with longstanding persistent AF was excluded from the overall regression analysis. CI, confidence interval

Recurrence of any atrial tachyarrhythmia was reported in 109 (38.9%) patients at 12 months follow‐up, more frequently in the non‐PV target (48.6% vs. 29.3%, *p* = .001) (Table 2). Covariates in the multivariate model that changed the exposure coefficient >10% for any atrial tachyarrhythmia recurrence included age, LA dimension and AF type. After adjusting, the difference in any atrial tachyarrhythmia recurrence between non‐PV target and PV target ablation showed a similar trend, but was not statistically significant (odds ratio [OR]: 1.50; 95% confidence interval [CI]: 0.86 – 2.62; *p* = .154) (Figure 1A). AF recurrence was observed in 82 (29.3%) patients, more frequently in the non‐PV target group (36.4% vs. 22.1%). Age, LA dimension, AF type, and hypertension resulted in a >10% change in exposure estimate. No statistically significant difference in AF recurrence was observed between the non‐PV target and the PV target ablation groups after adjusting (OR: 1.21; 95% CI: 0.66–2.20; *p* = .544) (Figure 1B). In total 47 (16.8%) patients had an AT recurrence during follow‐up, with a higher incidence in the non‐PV target group (22.9%) than the PV target group (10.7%) (*p* = .011). The cryoballoon ablation modality in the first ablation was the only covariate included in the multivariate model of which effect remained statistically significant after adjustment (OR: 2.19, 95% CI: 1.18–4.42; *p* = .023) (Figure 1C).

### 3.5 Sensitivity analysis

Logistic regression with inverse propensity weighting was performed in 267 patients (non‐PV target *n* = 135, PV target *n* = 132) and demonstrated outcomes on any atrial tachyarrhythmia, AT, and AF recurrence in line with the multivariate analysis presented in Figure 1. As compared to PV target ablation, patients who were treated with non‐PV target ablation had a nonstatistically significant trend toward a higher risk for any atrial tachyarrhythmia recurrence (OR: 1.62; 95% CI: 0.96–2.75; *p* = .074) and had a statistically significant higher risk for AT recurrence (OR: 2.39; 95% CI: 1.19–5.11; *p* = .019). There was no statistically significant difference in AF recurrence risk between patients who underwent non‐PV target ablation and patients who underwent PV target ablation (OR: 1.37; 95% CI: 0.77–2.46; *p* = .289).

### 3.6 Effect of non‐PV target ablation in patients with reconnected PVs

In 208 (74.3%) patients, one or more PV reconnections were identified during electrophysiological mapping at the repeat AF ablation procedure. Of these, 69 and 139 patients underwent non‐PV target and PV target ablation, respectively. After adjustment for confounding covariates, there was no significant difference in the number of reported atrial tachyarrhythmias (OR: 1.07; 95% CI: 0.51–2.17; *p* = .864) or AF (OR: 0.84; 95% CI: 0.37–1.86; *p* = 0. 681) recurrences between the two ablation strategies (Figure 1A/B). Patients undergoing non‐PV target ablation showed a trend for higher AT recurrence risk than patients with PV target ablation (OR: 2.38; 95% CI: 1.00–5.80; *p* = .051) after adjustment for chronic obstructive pulmonary disease and the use of contact force‐sensing catheters. LA size and body mass index were excluded from the model that resulted in a lower AIC (Figure 1C).

### 3.7 Effect of isolated PVs

**Figure 2:**
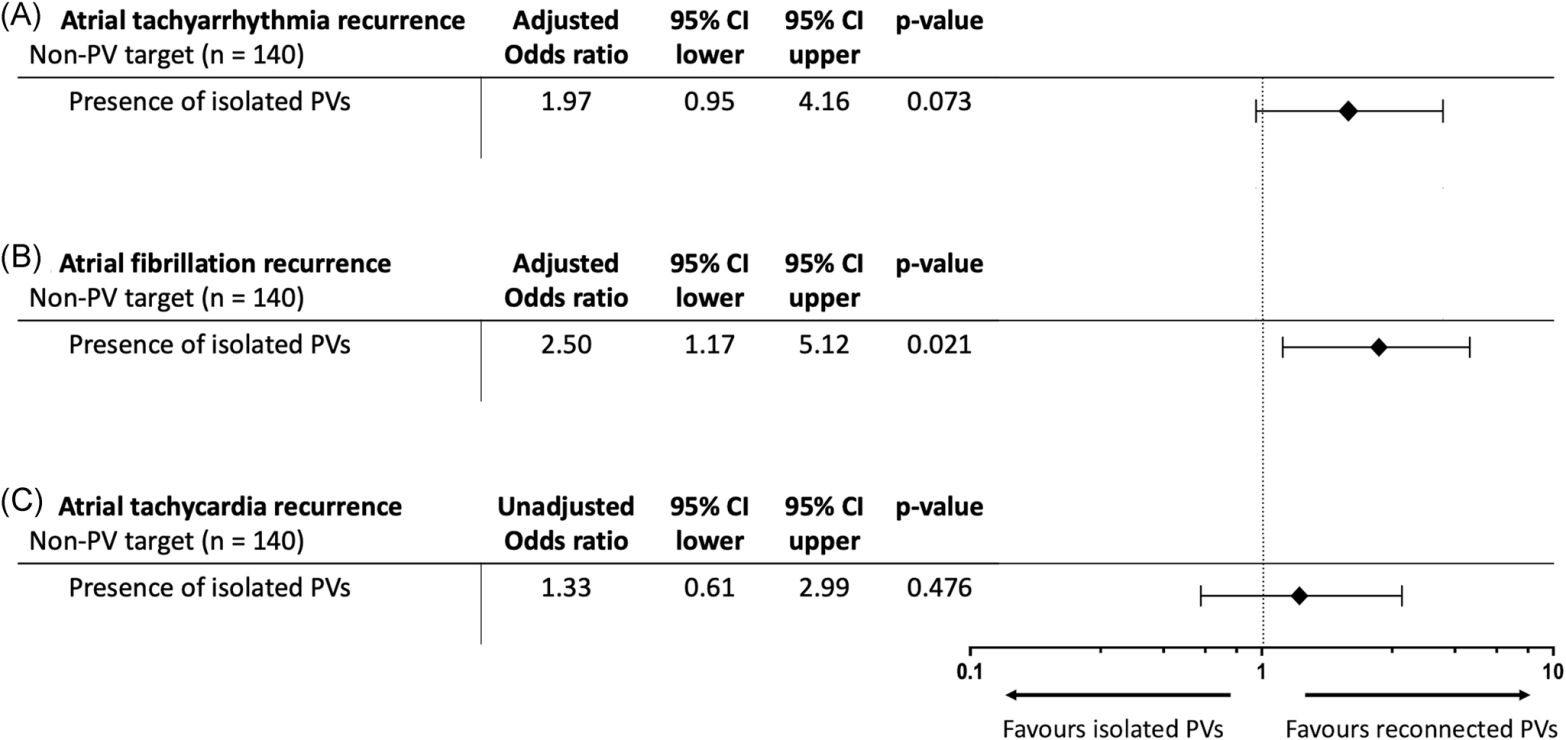
Arrhythmia recurrence risk of patients with isolated pulmonary veins (PV) who underwent non‐PV target ablation. This figure presents the atrial tachyarrhythmia (A), atrial fibrillation (B), and atrial tachycardia (C) recurrence risk. Here, the odds ratio displays the arrhythmia recurrence risk of patients with isolated PV who underwent non‐PV target ablation versus patients with reconnected PVs who underwent non‐PV target ablation. Non‐PV target ablation was defined as PV re‐isolation with additional left atrial ablation. Of note, we only performed an unadjusted analysis for atrial tachycardia recurrence because of limited statistical degrees of freedom in this analysis. CI, confidence interval

Patients with isolated PVs before repeat ablation showed a higher atrial tachyarrhythmia recurrence rate (55%) than patients with PV reconnections (33.2%) (*p* = .001). We performed subanalyses to determine the arrhythmia recurrence risk of patients with isolated PVs before repeat AF ablation who underwent non‐PV target ablation compared to patients with reconnected PVs who underwent non‐PV target ablation (Figure 2). We found that, after adjustment for covariates, LA size, use of contact force‐sensing catheters, and posterior wall isolation, patients with isolated PVs who underwent non‐PV target ablation had a nonsignificant trend toward higher risks for any atrial tachyarrhythmia recurrences (OR: 1.97; 95% CI: 0.95–4.16; *p* = .073) (Figure 2A). Additionally, patients with isolated PVs who underwent non‐PV target ablation had a significantly higher risk for AF recurrence (OR: 2.50; 95% CI: 1.17–5.12; *p* = .021) after adjustment for cryoballoon ablation during index procedure, non‐vitamin K oral anticoagulants or calcium antagonist use, and posterior wall isolation (Figure 2B). To avoid overfitting, the use of contact force‐sensing catheters was excluded from that model. Lastly, we observed no differences between groups for AT recurrences (unadjusted OR: 1.33; 95% CI: 0.61–2.99; *p* = .476. Note, we only performed an unadjusted analysis because of limited statistical degrees of freedom in this analysis (Figure 2C).

### 3.8 AAD use

**Figure 3:**
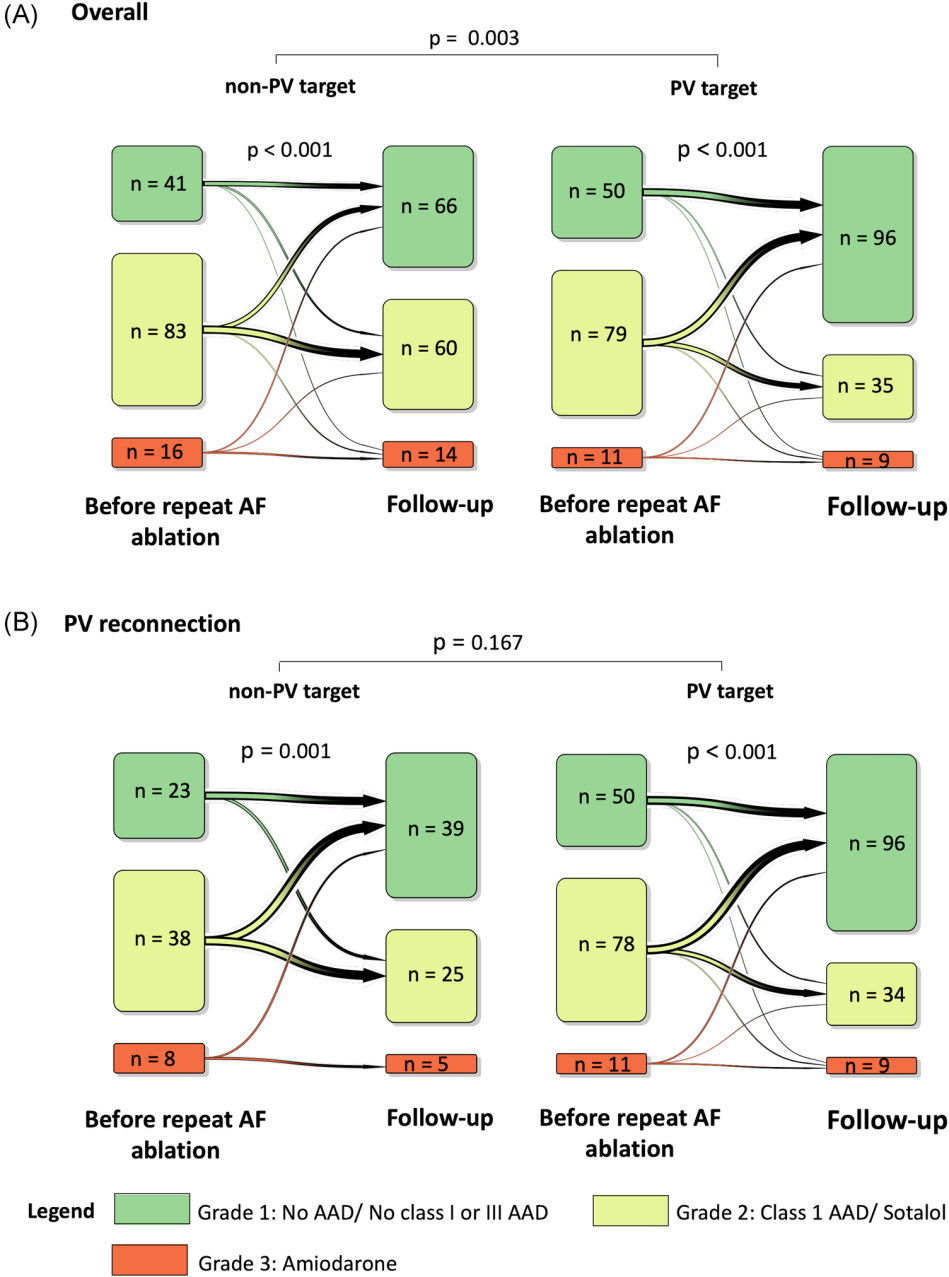
Antiarrhythmic drug (AAD) grades before repeat atrial fibrillation (AF) ablation and follow‐up. We defined AAD use according to the following grades: Grade 1 (green) included patients who did not use a Class I or Class III AAD, but Class II, Class IV, or no AAD instead, Grade 2 (yellow) included patients who used Class I AAD or sotalol and Grade 3 (red) compromised patients using amiodarone. (A) AAD grade before repeat AF ablation and at follow‐up of all patients who underwent non‐pulmonary vein (PV) and PV target ablation, and group comparison. (B) AAD grade before and at follow‐up of patients with reconnected PVs who underwent non‐PV target and PV target ablation and group comparison. Non‐PV target ablation was defined as PV reisolation with additional left atrial ablation, and PV target ablation as PV reisolation with or without wide antrum circumferential ablation

**Figure 4:**
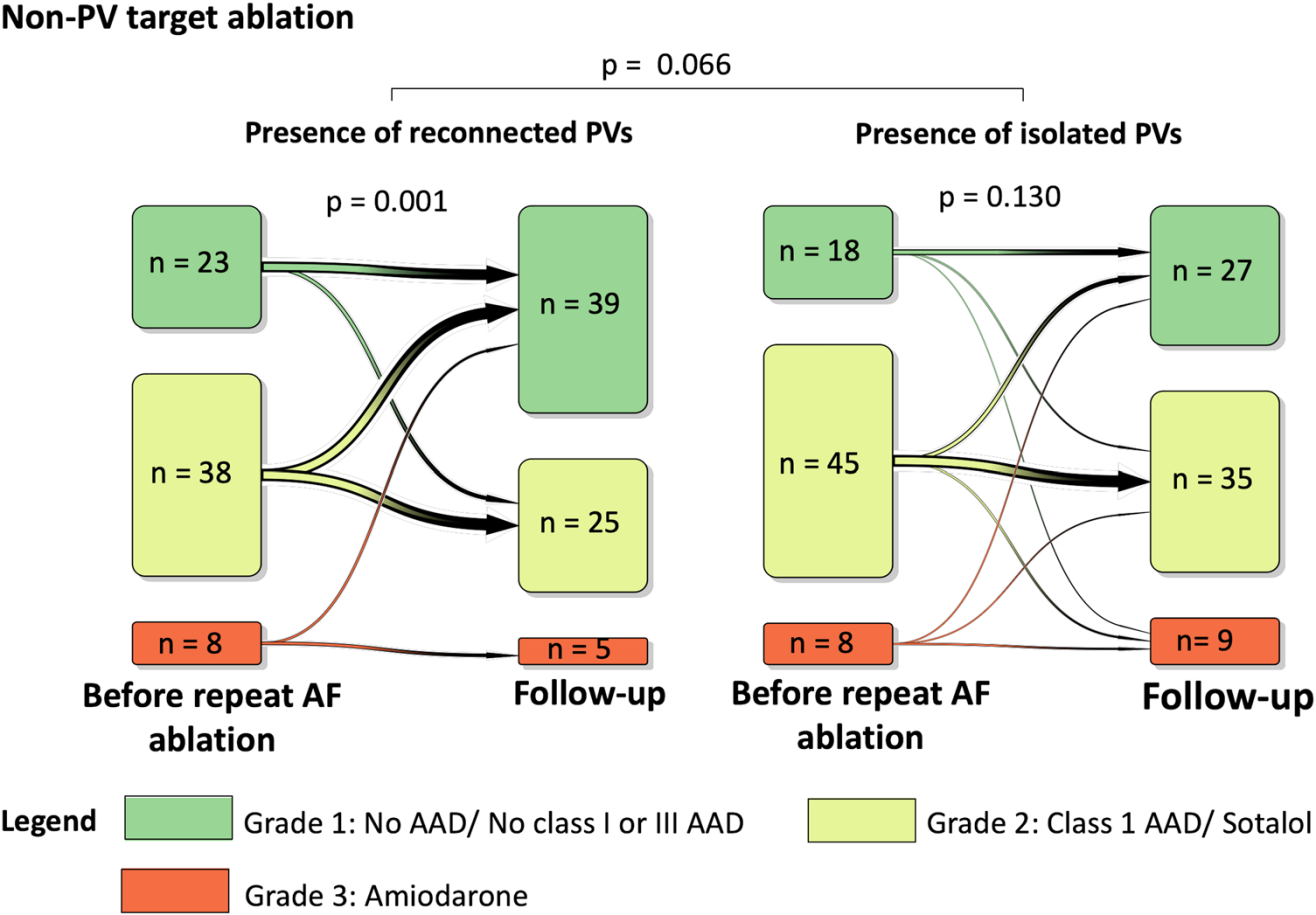
Antiarrhythmic drug (AAD) grades before repeat atrial fibrillation (AF) ablation and follow‐up. We defined AAD use according to the following grades: Grade 1 (green) included patients who did not use a Class I or Class III AAD, but Class II, Class IV, or no AAD instead, Grade 2 (yellow) included patients who used Class I AAD or sotalol, and Grade 3 (red) compromised patients using amiodarone. Here, we present the AAD grade in patients with isolated pulmonary veins (PVs) and reconnected PVs who underwent non‐PV target ablation and group comparison. Non‐PV target ablation was defined as PV reisolation with additional left atrial ablation

A significant de‐escalation of AAD therapy (defined as discontinuation of AAD or use of lower grades of AAD) was observed in both groups (*p* < .001; Figure 3A). Overall, PV target ablation resulted in a more profound de‐escalation of AAD therapy than non‐PV target ablation (*p* = .003; Figure 3A). However, there was no statistically significant difference in AAD therapy de‐escalation between non‐PV target and PV target ablation in patients with reconnected PVs (*p* = .167; Figure 3B). Patients with isolated PVs who underwent non‐PV target ablation AAD therapy was not de‐escalated (*p* = .130; Figure 4). Also, we observed no significant difference in the AAD therapy de‐escalation between patients with reconnected and isolated PVs who underwent non‐PV target ablation (*p* = .066; Figure 4).

### 3.9 AF‐related symptoms

EHRA scores improved from 2.47 ± 0.62 before repeat ablation to 1.66 ± 0.77 after 12 months follow‐up (*p* < .001). This improvement was more evident in patients who underwent PV target ablation (2.36 ± 0.63 before vs. 1.47 ± 0.64 12 months after repeat ablation, *p* < .001), in comparison with patients who underwent non‐PV target ablation (2.58 ± 0.6 vs. 1.86 ± 0.84; before repeat ablation and at follow‐up, *p* < .001) (group comparison *p* = .003). Similar findings were observed in patients with reconnected PVs before repeat AF ablation, both groups improved significantly in EHRA class (*p* < .001) and the improvement was more evident in patients who underwent PV target ablation (group comparison *p* = .014). Also, patients with PV reconnection at repeat ablation undergoing non‐PV target ablation demonstrated a greater improvement in the EHRA score (2.54 ± 0.61 before repeat ablation vs. 1.62 ± 0.73 at follow‐up, *p* < .001), in comparison with patients with isolated PVs (2.63 ± 0.59 before ablation and 2.08 ± 0.59 at follow‐up, *p* = .001) (group comparison *p* < .001).

## 4 DISCUSSION

In this multicentre retrospective study, patients suffered significantly higher recurrences of atrial tachyarrhythmia, AF, and AT after non‐PV target ablation compared to those undergoing PV target ablation. After adjustment for covariates, the risk for atrial arrhythmia or AF recurrences was not statistically significant anymore. Hence, the risk of atrial tachyarrhythmia recurrence was strongly driven by baseline characteristics. However, AT recurrence risk for patients after non‐PV target ablation remained statistically significant. In addition, most patients were able to step down in level of AAD after repeat ablation, but not those with isolated PVs and non‐PV targeted ablation. Patients in both groups showed significant improvement of AF‐related symptoms, more evident in the PV target group.

### 4.1 Repeat AF ablation

Freedom of atrial tachyarrhythmias after repeat ablation ranges between 50% and 75% depending on patient characteristics, ablation strategy, and the presence of reconnected PVs.^14^, ^15^, ^16^, ^18^ The comparative efficacy of non‐PV target ablation in the setting of repeat ablation (as compared to the first ablation) is relatively unexplored. Fichtner et al.^15^ demonstrated no additional value of an anterior ablation line in addition to PV reisolation compared to PV reisolation alone at repeat ablation. In addition, another study failed to identify an association between non‐PV target ablation and arrhythmia outcomes, and there was no difference in atrial tachyarrhythmia or AF recurrence risk.^16^ More recently, Pothineni et al.^22^ retrospectively included 196 patients who underwent repeat AF ablation. Reisolation was performed in 93 patients, and 103 patients underwent a posterior wall isolation ± PV reisolation. The authors conclude that posterior wall isolation did not improve arrhythmia‐free survival. However, in line with the findings of our study, patients who underwent posterior wall isolation were older, had more frequently hypertension, and persistent AF. The presence and ablation therapy of non‐PV triggers during repeat AF ablation was associated with worse arrhythmia‐free survival as compared to patients without non‐PV triggers.^23^ Authors have shown that 33% of the newly identified non‐PV triggers during repeat ablation were at non‐PV target sites of the first AF ablation.^23^ Indeed, it remains unestablished what the arrhythmia‐free survival would have been if PV trigger ablation was not performed in these patients.^23^

### 4.2 The role of PVs

In our study, patients with reconnected PVs at repeat non‐PV target ablation had better outcomes compared to patients with isolated PVs, suggesting that occurrence and maintenance of atrial tachyarrhythmias are PV‐dependent in many patients. In an observational cohort study for 143 subjects with first repeat AF ablation, patients with reconnected PVs similarly showed a better atrial arrhythmia‐free survival than patients with isolated PVs.^14^ Another study investigated the role of the PV reconnection during repeat AF ablation in patients who had an atrial tachyarrhythmia relapse >36 months after initial PV isolation.^17^ Although PV reconnection was observed in 81% of the patients, that study suggests that a repeat ablation strategy should also involve substrate ablation regardless of PV reconnection.^17^ However, there was no clear comparison in clinical outcome between patients with reconnected PV and isolated PVs.^17^ In contrast with our study, other authors did not find a difference in atrial tachyarrhythmia‐free survival between patients with or without reconnected PV who underwent posterior wall isolation during repeat AF ablation.^22^ Although we did not observe a difference in atrial tachyarrhythmia recurrence risk after adjustment, we found a higher risk for AF recurrences in patients with isolated PVs undergoing non‐PV target ablation.

In the RACE‐AF trial, all patients underwent a repeat procedure 4–6 months after the first AF ablation.^2^ Twenty percent of the patients with isolated PVs had AF recurrences, despite a 96% reduction of AF burden in these patients.^2^ In our study, we found that 56% of patients with non‐PV target ablation and isolated PVs reported an atrial tachyarrhythmia recurrence. Our study does not provide data on burden reduction. However, we observed no de‐escalation in AAD therapy and the slightest improvement in AF‐related symptoms in patients with isolated PVs who underwent non‐PV target ablation.

### 4.3 Non‐PV target ablation therapy during first AF ablation

Most of the non‐PV target ablation strategies data are derived from the first ablation procedures. We believe that the findings of the studies during the first AF ablation discussed below support our data in the setting of repeat AF ablation. In our study, 90 patients in the non‐PV target ablation group underwent posterior wall isolation. A previously meta‐regression study by Sau et al.^10^ showed a 19% reduction in AF risk following posterior wall isolation during the first AF ablation. However, this meta‐regression study included only three randomized trials, and the studies had different comparators.^10^ More recently, Lee et al.^11^ randomized 217 patients with persistent AF to either posterior wall isolation or PV isolation alone. They observed no difference in arrhythmia‐free survival after a median follow‐up of 16 months. The authors further found the absence of isolation of the posterior wall in 50% of the patients who underwent a repeat AF ablation.^11^ The STAR AF II trial demonstrated that linear or CFAE ablation did not improve arrhythmia‐free survival compared to PV isolation alone in patients with persistent AF.^13^ In addition, patients who underwent linear LA ablation had a statistically significant higher atrial tachyarrhythmia recurrence rate as compared to patients who underwent PV isolation alone.^5^

Magnetic resonance imaging‐identified LA tissue fibrosis has been associated with more atrial arrhythmia recurrence.^24^ In addition, Masuda et al.^12^ created an electroanatomical map before the PV isolation in 403 patients with paroxysmal AF. Upon identification of low voltage areas, patients were either treated by PV isolation + low voltage area ablation or PV isolation alone, and patients without low voltage areas who underwent PV isolation were included as a third comparison group.^12^ It was found that patients with low voltage areas had more atrial arrhythmia recurrences compared to patients without low voltage areas. Ablation of these areas, however, failed to improve long‐term outcomes.^12^ The DECAAF II trial recently confirmed the relation between MRI‐detected atrial fibrosis and ablation outcome, but could not demonstrate a benefit of ablation of fibrosis areas.^25^

Taken together, there is no striking evidence in the literature that additional non‐PV target ablation during first or repeat AF ablation improves outcomes compared to PV target ablation alone. Supported by these data, we believe that the role of the PVs is crucial, and extensive LA ablation should be used only in a selected patient with extra caution. The mechanism of AF beyond the PVs is complex, warranting further investigation to confirm the efficacy of tailored AF ablation approaches in patients with isolated PVs. Besides, a remaining question is what a realistic therapeutic goal is in patients with extensive fibrosis or isolated PVs.

### 4.4 Study limitations

This study has some limitations. First, only patients were included who underwent repeat AF ablation, and the admission to that therapy was based on a shared decision between patient and operator. Therefore, patients who did not undergo repeat AF ablation despite having an atrial tachyarrhythmia recurrence after a first ablation procedure were excluded, and our results are representative only for patients actually undergoing repeat ablation. Second, the ablation strategy was performed at the operators' discretion and differences in baseline characteristics were observed between both ablation strategies. Therefore, adjustment was performed for covariates that changed the exposure coefficient to >10%. Thus, baseline characteristics that were associated with an ablation strategy were included in the multivariate model. Also, a sensitivity analysis was performed with inverse propensity weighting to assess the robustness of the multivariate model and demonstrate comparable outcomes. However, we were unable to correct for electrophysiological findings, as the electroanatomical mapping data were unavailable. Nevertheless, the notable differences in atrial tachyarrhythmia recurrence between groups provide important insights for daily practice. The actual efficacy of non‐PV target ablation should be investigated in randomized trials. It is also important to reconsider acceptable outcomes in terms of arrhythmia recurrences and patient satisfaction in patients undergoing non‐PV target ablation. The current consensus of the absence of >30 s atrial tachyarrhythmia is likely very strict and ignores symptomatic improvement. Third, the non‐PV targets strategies were a “mixed bag” of LA ablation and this study was underpowered to determine the effect of each ablation site. Fourth, none of the patients included in this study underwent AF trigger ablation. Another limitation of this study is that routine follow‐up instead of a structured study follow‐up scheme was used. Nonetheless, follow‐up visits and monitoring were the same in both groups.

## 5 CONCLUSION

In patients undergoing first repeat AF ablation, non‐PV target ablation failed to improve arrhythmia free survival compared to PV target approaches. Atrial tachyarrhythmia, AF, and AT recurrences were more frequently observed in patients undergoing non‐PV target ablation. These differences were partially driven by the baseline characteristics of patients. However, patients with a non‐PV target ablation remained at higher risk for AT recurrence after adjustment for differences in baseline characteristics. Therefore, randomized controlled trials are needed to determine the actual effect of non‐PV target ablation during repeat AF ablations.

## CONFLICTS OF INTEREST

Daniel Mol reports speaker/consultancy fee from Abbott. Giovanni J. M. Tahapary reports speaker fees from Medtronic. Joris R. de Groot reports research grants to his institution from Abbott, Atricure Inc., Bayer, Boston Scientific, Daiichi Sankyo, Johnson & Johnson, Medtronic, consultancy/speaker fees from Abbott, AtriAN Medical, Atricure Inc., Bayer, Biotronik, CVOI, Daiichi Sankyo, IPP Med, Itreas, Medtronic, Novartis, Servier, outside the submitted work. Jonas S. S. G. de Jong reports consultancy fees from Medtronic and speaker fees from Daiichi‐Sankyo and Bayer, outside the submitted work.

## Supporting information

Supporting information.

## Data Availability

Data underlying this article will be shared on reasonable request. Proposals should be directed to the corresponding author.

## References

[1] Haissaguerre M , Jais P , Shah DC , et al. Spontaneous initiation of atrial fibrillation by ectopic beats originating in the pulmonary veins. N Engl J Med. 1998;339:659‐666. 9725923 10.1056/NEJM199809033391003

[2] Sørensen SK , Johannessen A , Worck R , Hansen ML , Hansen J . Radiofrequency vs. Cryoballoon catheter ablation for paroxysmal atrial fibrillation: durability of pulmonary vein isolation and effect on AF burden: the RACE‐AF Randomized Controlled Trial. Circ Arrhythm Electrophysiol. 2021;14:009573. 10.1161/CIRCEP.120.009573 PMC8136462 33835823

[3] Packer DL , Mark DB , Robb RA , et al. Effect of catheter ablation vs antiarrhythmic drug therapy on mortality, stroke, bleeding, and cardiac arrest among patients with atrial fibrillation: the CABANA Randomized Clinical Trial. JAMA. 2019;321:1261‐1274. 30874766 10.1001/jama.2019.0693 PMC6450284

[4] Kuck KH , Brugada J , Furnkranz A , et al. Cryoballoon or radiofrequency ablation for paroxysmal atrial fibrillation. N Engl J Med. 2016;374:2235‐2245. 27042964 10.1056/NEJMoa1602014

[5] Andrade JG , Champagne J , Dubuc M , et al. Cryoballoon or radiofrequency ablation for atrial fibrillation assessed by continuous monitoring: a Randomized Clinical Trial. Circulation. 2019;140:1779‐1788. 31630538 10.1161/CIRCULATIONAHA.119.042622

[6] Bordignon S , Furnkranz A , Perrotta L , et al. High rate of durable pulmonary vein isolation after second‐generation cryoballoon ablation: analysis of repeat procedures. Europace. 2015;17:725‐731. 25618741 10.1093/europace/euu331

[7] Mol D , Houterman S , Balt JC , et al. Complications in pulmonary vein isolation in the Netherlands Heart Registration differ with sex and ablation technique. Europace. 2020;23:216‐225. 10.1093/europace/euaa255 33141152

[8] Cheung CC , Deyell MW , Macle L , et al. Repeat atrial fibrillation ablation procedures in the CIRCA‐DOSE Study. Circ Arrhythm Electrophysiol. 2020;13:e008480. 32701361 10.1161/CIRCEP.120.008480

[9] Okamatsu H , Okumura K . Strategy and outcome of catheter ablation for persistent atrial fibrillation—impact of progress in the mapping and ablation technologies. Circ J. 2017;82:2‐9. 29187667 10.1253/circj.CJ-17-1205

[10] Sau A , Al‐Aidarous S , Howard J , et al. Optimum lesion set and predictors of outcome in persistent atrial fibrillation ablation: a meta‐regression analysis. Europace. 2019;21:1176‐1184. 31071213 10.1093/europace/euz108 PMC6680367

[11] Lee JM , Shim J , Park J , et al. The Electrical Isolation of the Left Atrial Posterior Wall in Catheter Ablation of Persistent Atrial Fibrillation. JACC Clin Electrophysiol. 2019;5:1253‐1261. 31753429 10.1016/j.jacep.2019.08.021

[12] Masuda M , Asai M , Iida O , et al. Additional low‐voltage‐area ablation in patients with paroxysmal atrial fibrillation: results of the Randomized Controlled VOLCANO Trial. J Am Heart Assoc. 2020;9:e015927. 32578466 10.1161/JAHA.120.015927 PMC7670527

[13] Verma A , Jiang CY , Betts TR , et al. Approaches to catheter ablation for persistent atrial fibrillation. N Engl J Med. 2015;372:1812‐1822. 25946280 10.1056/NEJMoa1408288

[14] Kim TH , Park J , Uhm JS , Joung B , Lee MH , Pak HN . Pulmonary vein reconnection predicts good clinical outcome after second catheter ablation for atrial fibrillation. Europace. 2017;19:961‐967. 27256420 10.1093/europace/euw128

[15] Fichtner S , Sparn K , Reents T , et al. Recurrence of paroxysmal atrial fibrillation after pulmonary vein isolation: Is repeat pulmonary vein isolation enough? A prospective, randomized trial. Europace. 2015;17:1371‐1375. 25694536 10.1093/europace/euu389

[16] Daimee UA , Akhtar T , Boyle TA , et al. Repeat catheter ablation for recurrent atrial fibrillation: electrophysiologic findings and clinical outcomes. J Cardiovasc Electrophysiol. 2021;32:628‐638. 33410561 10.1111/jce.14867

[17] Shah S , Barakat AF , Saliba WI , et al. Recurrent atrial fibrillation after initial long‐term ablation success: electrophysiological findings and outcomes of repeat ablation procedures. Circ Arrhythm Electrophysiol. 2018;11:e005785. 29654129 10.1161/CIRCEP.117.005785

[18] Calkins H , Hindricks G , Cappato R , et al. 2017 HRS/EHRA/ECAS/APHRS/SOLAECE expert consensus statement on catheter and surgical ablation of atrial fibrillation. Heart Rhythm. 2017;14:e275‐e444. 28506916 10.1016/j.hrthm.2017.05.012 PMC6019327

[19] Nademanee K , McKenzie J , Kosar E , et al. A new approach for catheter ablation of atrial fibrillation: mapping of the electrophysiologic substrate. J Am Coll Cardiol. 2004;43:2044‐2053. 15172410 10.1016/j.jacc.2003.12.054

[20] Lang RM , Badano LP , Mor‐Avi V , et al. Recommendations for cardiac chamber quantification by echocardiography in adults: an update from the American Society of Echocardiography and the European Association of Cardiovascular Imaging. Eur Heart J Cardiovasc Imaging. 2015;16:233‐270. 25712077 10.1093/ehjci/jev014

[21] VanderWeele TJ . Principles of confounder selection. Eur J Epidemiol. 2019;34:211‐219. 30840181 10.1007/s10654-019-00494-6 PMC6447501

[22] Pothineni NVK , Lin A , Frankel DS , et al. Impact of left atrial posterior wall isolation on arrhythmia outcomes in patients with atrial fibrillation undergoing repeat ablation. Heart Rhythm O2. 2021;2:489‐497. 34667964 10.1016/j.hroo.2021.07.004 PMC8505210

[23] Kim D , Hwang T , Kim M , et al. Extra‐pulmonary vein triggers at de novo and the repeat atrial fibrillation catheter ablation. Front Cardiovasc Med. 2021;8:759967. 34805314 10.3389/fcvm.2021.759967 PMC8600078

[24] Marrouche NF , Wilber D , Hindricks G , et al. Association of atrial tissue fibrosis identified by delayed enhancement MRI and atrial fibrillation catheter ablation: the DECAAF study. JAMA. 2014;311:498‐506. 24496537 10.1001/jama.2014.3

[25] Nicholls M . Diverse topics tackled by latest ‘hot’ research. Eur Heart J. 2021;42(44):4512‐4514. 10.1093/eurheartj/ehab685 34739056

