# Supplementary material for: Strategies for repeat ablation for atrial fibrillation: A multicentre comparison of nonpulmonary vein versus pulmonary vein target ablation
Source: J Cardiovasc Electrophysiol. 2022 Mar 22;33(5):885–96. doi: 10.1111/jce.15441 (PMC9315029; doi:10.1111/jce.15441)
Supplement: Supplementary file 1 — Supporting information. [file JCE-33-885-s001.docx]

| **Table 1: Ablation strategies** | Overall  (n = 280) |
| --- | --- |
| **Non-PV target ablation** |  |
| PWI | 24 (8.6) |
| PWI + CFAE | 12(4.3) |
| PWI + MIL | 6 (2.1) |
| PWI + MIL + CFAE | 6 (2.1) |
| PWI + WACA + MIL | 2 (0.7) |
| PWI + WACA + CFAE | 2 (0.7) |
| PWI + WACA | 1 (0.4) |
| PWI + WACA + MIL +CFAE | 1 (0.4) |
| Re-isolation + PWI | 15 (5.4) |
| Re-isolation + CFAE | 12 (4.3) |
| Re-isolation + MIL + roofline | 9 (3.2) |
| Re-isolation + PWI + CFAE | 7 (2.5) |
| Re-isolation + PWI + MIL | 5 (1.8) |
| Re-isolation + PWI + WACA + CFAE | 5 (1.8) |
| Re-isolation + MIL | 3 (1.1) |
| Re-isolation + MIL + CFAE | 3 (1.1) |
| Re-isolation + PWI + MIL + CFAE | 2 (0.7) |
| Re-isolation + WACA + CFAE | 2 (0.7) |
| Re-isolation + roofline | 2 (0.7) |
| Re-isolation + PWI + WACA | 1 (0.4) |
| Re-isolation + PWI + WACA + MIL | 1 (0.4) |
| Re-isolation + roofline + CFAE | 1 (0.4) |
| Re-isolation + MIL + roofline + CFAE | 1 (0.4) |
| CFAE | 3 (1.1) |
| Roofline | 3 (1.1) |
| Roofline + CFAE | 2 (0.7) |
| Roofline + WACA | 1 (0.4) |
| MIL + CFAE | 3 (1.1) |
| MIL + roofline | 2 (0.7) |
| MIL + roofline + CFAE | 2 (0.7) |
| MIL | 1 (0.4) |
| **PV target ablation** |  |
| WACA | 1 (0.4) |
| Re-isolation + WACA | 24 (8.6) |
| Re-isolation | 115 (41.1) |

Ablation strategies used during non-pulmonary vein (PV) target and PV target ablation. Posterior wall isolation (PWI), complex fractionated atrial electrogram ablation (CFAE), mitral isthmus line (MIL), and wide atrium circumferential ablation (WACA). Number (%)
